# Indole and Benzimidazole Bichalcophenes: Synthesis, DNA Binding and Antiparasitic Activity

**DOI:** 10.1016/j.ejmech.2017.10.056

**Published:** 2018-01-01

**Authors:** Abdelbasset A. Farahat, Mohamed A. Ismail, Arvind Kumar, Tanja Wenzler, Reto Brun, Ananya Paul, W. David Wilson, David W. Boykin

**Affiliations:** a Department of Chemistry, Georgia State University, Atlanta, GA, 30303, United States; b Department of Pharmaceutical Organic Chemistry, Faculty of Pharmacy, Mansoura University, Mansoura 35516, Egypt; c Department of Chemistry, Faculty of Science, Mansoura University, Mansoura 35516, Egypt; d Swiss Tropical and Public Health Institute, 4002 Basel, Switzerland; e University of Basel, 4003 Basel, Switzerland

**Keywords:** Diamidines, Stille coupling, Pinner reaction, DNA minor groove binders, Trypanocidal, Antimalarial, African sleeping sickness, Malaria

## Abstract

A novel series of indole and benzimidazole bichalcophene diamidine derivatives were prepared to study their antimicrobial activity against the tropical parasites causing African sleeping sickness and malaria. The dicyanoindoles needed to synthesize the target diamidines were obtained through Stille coupling reactions while the bis-cyanobenzimidazoles intermediates were made *via* condensation/cyclization reactions of different aldehydes with 4-cyano-1,2-diaminobenzene. Different amidine synthesis methodologies namely, lithium bis-trimethylsilylamide (LiN[Si(CH3)_3_]_2_) and Pinner methods were used to prepare the diamidines. Both types (indole and benzimidazole) derivatives of the new diamidines bind strongly with the DNA minor groove and generally show excellent *in vitro* antitrypanosomal activity. The diamidino-indole derivatives also showed excellent *in vitro* antimalarial activity while their benzimidazole counterparts were generally less active. Compound **7c** was highly active *in vivo* and cured all mice infected with *Trypanosoma brucei rhodesiense*, a model that mimics the acute stage of African sleeping sickness, at a low dose of 4 × 5 mg/kg i.p. and hence **7c** is more potent *in vivo* than pentamidine.

## Highlights

• A novel series of indole- and benzimidazole bichalcophene diamidine derivatives were prepared.
• The new diamidines exhibited very strong DNA binding as seen from the ΔTm values.
• These diamidines are shown to be DNA minor groove binders as seen from the CD spectra.
• Both dicationic indole and benzimidazole derivatives showed very good *in vitro* antitrypanosomal and antimalarial activity.
• Compound **4c** was highly efficacious *in vivo* curing all T. b. r. infected mice.

## Graphical abstract

**Figure:**
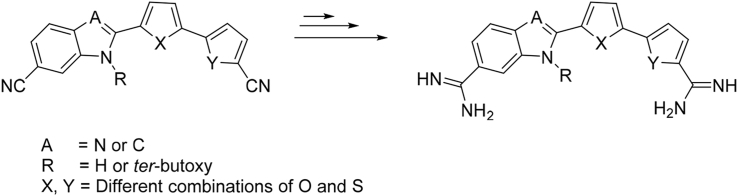

## 1 Introduction

*Trypanosoma brucei* subspecies and *Plasmodium falciparum*, pathogenic single celled parasites, are the infective agents which cause human African trypanosomiasis (sleeping sickness) and malaria. African trypanosomes are transmitted by the blood-sucking tsetse fly and threaten about 65 million humans in the poorest regions in the world within 36 countries. The estimated number of patients is currenlty below 20,000 due to extensive sleeping sickness control programmes [1]. Without an effective treatment, African sleeping sickness is generally lethal whereas the current drugs are still unsatisfactory [2]. Malaria is a mosquito-borne disease which is endemic in 91 countries worldwide. In 2016, the WHO estimated the malaria burden to 212 million cases and 429,000 deaths [3]. There are a number of drugs to treat malaria, however drug-resistance is an ever increasing problem. Recently, resistance has emerged also to the so far highly effective artemisinin and artemisinin derivatives which are part of the currently applied artemisinin based combination therapy (ACT) [4]. There is a clear need to identify new more effective and well tolerated drugs for both of these diseases.

**Fig. 1:**
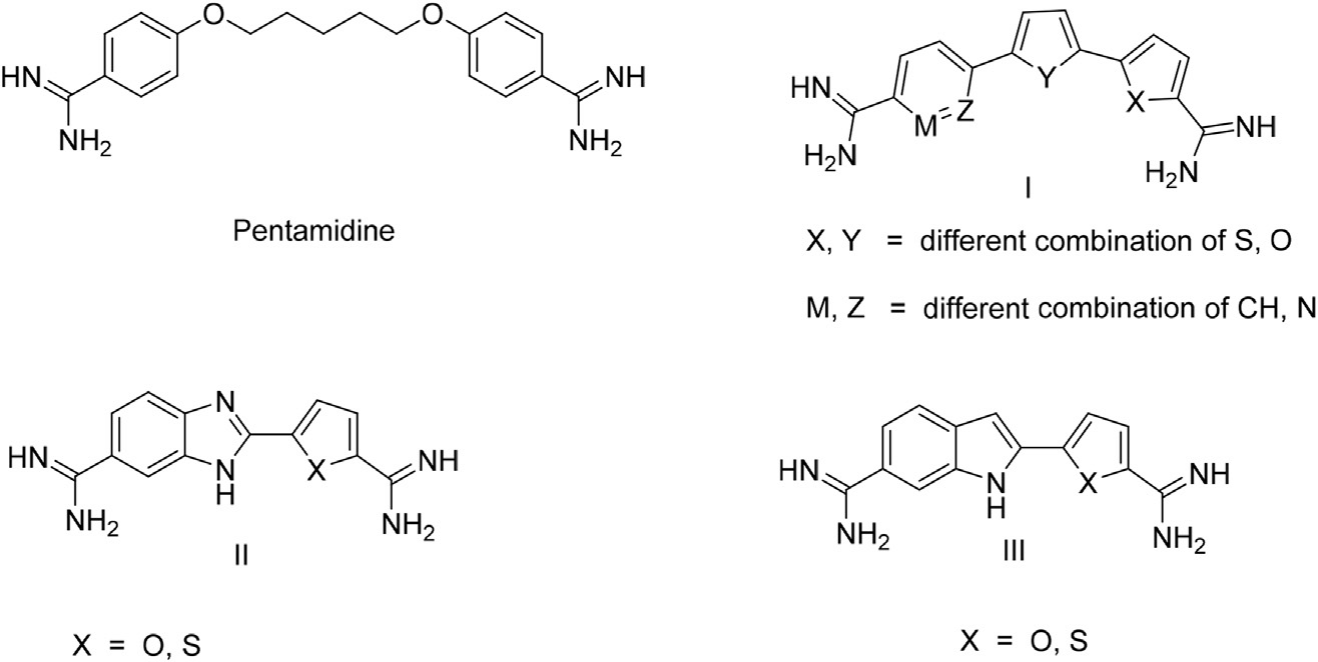
Antiparasitic diamidines.

Aryl diamidines, DNA minor groove binders, show promise against both parasites. Pentamidine (Fig. 1) is the only diamidine which has shown significant human use [5]. Pentamidine is the treatment of choice clinically for 1st stage *T. b. gambiense* infections since the 1930s and it is still effective [6]. Diminazene, is another diamidine with high antiprotozoal activity and it is in use to treat animal trypanosomiasis (nagana) since the 1950s [7].

We previously have reported some phenyl bichalcophenes (compound I, Fig. 1), that showed strong DNA binding and exhibited potent antiparasitic activity [8]. In a related investigation, we reduced the size of the molecule by removing one five member ring from compound I and replaced the 6 member ring with either a benzimidazole [9] or an indole [10] moiety (compound II and III, Fig. 1). Both approaches gave compounds that are highly active *in vitro* while the indole containing compounds resulted in superior *in vivo* activity compared to the benzimidazole containing ones.

In the present investigation, we report the synthesis of a new class of bichalcophenes where we replace the 6 membered aryl ring of compound I with either an indole or a benzimidazole moiety, this approach also involves adding one more 5 member heterocyclic ring to compounds of type II and III aiming for improving the antiprotozoal effectiveness of this class of compounds.

## 2 Results and discussion

### 2.1 Chemistry

**Scheme 1:**
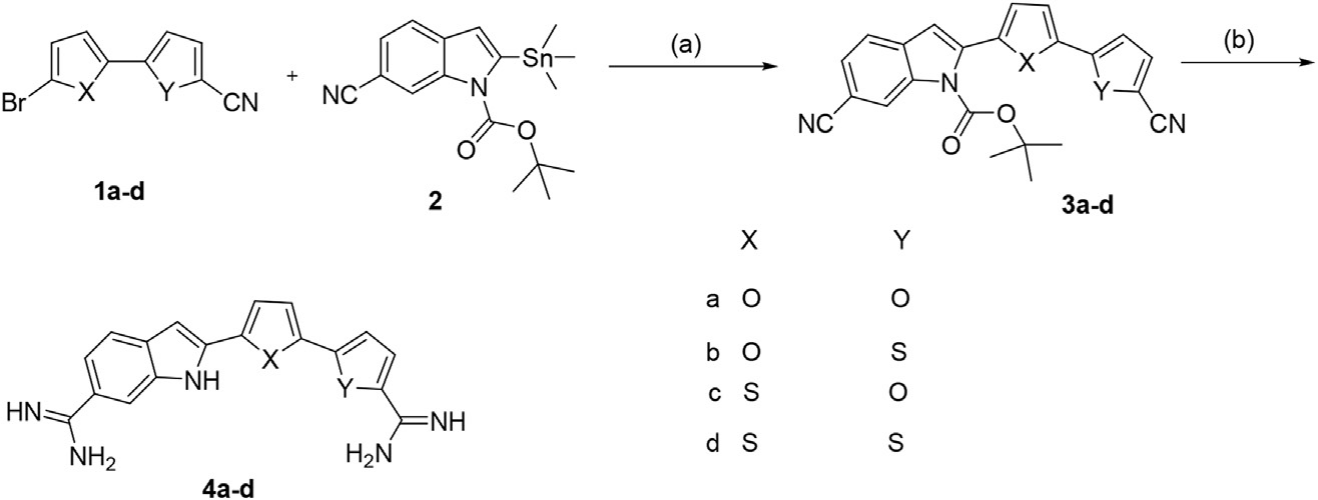
Reagenta and conditions: (a) Pd(PPh_3_), dioxane; (b) i-LiN(TMS)_2_/THF, ii-HCl gas/EtOH.

Synthesis of the indole-bichalcophene diamidine derivatives **4a-d** is outlined in Scheme 1. The bis-nitriles **3a-d** were prepared by Stille coupling [11], [12], [13] reaction between the bromo derivatives **1a-d** and 2-(trimethylstannyl)-1-(*tert*-butoxycarbonyl)- 1*H*-indole-6-carbonitrile **2** using palladium tetrakistriphenylphosphine as a catalyst. The bis-nitriles **3a-d** on reaction with a THF solution of lithium bis(trimethylsilyl)amide [14], followed by treatment with ethanolic HCl yielded the hydrochloride salts of the diamidines **4a-d**.

**Scheme 2:**
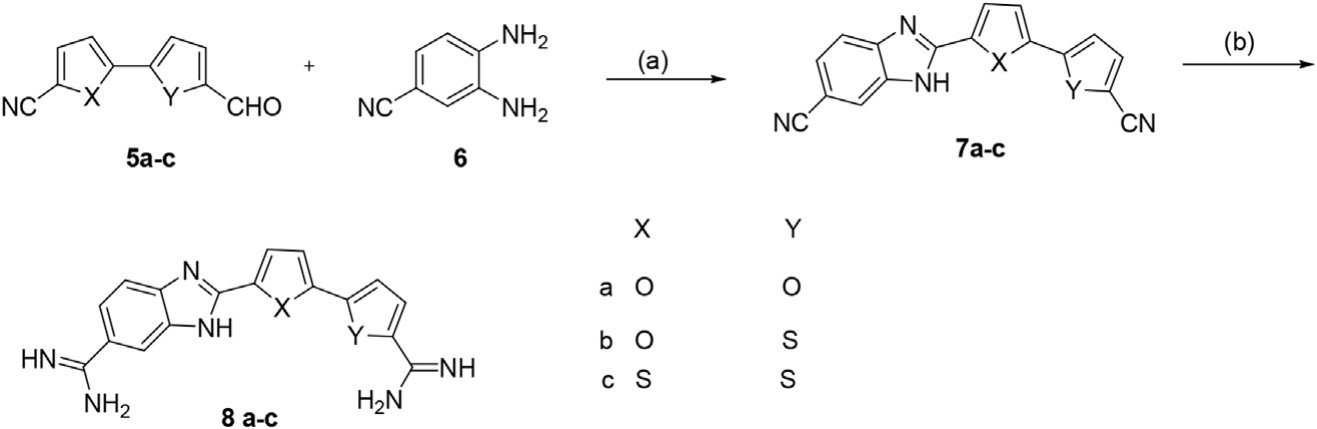
Reagenta and conditions: (a) 1,4-benzoquinone, EtOH, or Sodium bisulphite, DMF; (b) i-HCl gas/EtOH, ii-NH_3_ gas.

Scheme 2, illustrates the synthetic approach for the new benzimidazole-bichalcophene derivatives **8a-c**. The aldehydes **5a-c** underwent oxidative-condensation with 3,4-diaminobenzonitrile **6** using either sodium bisulphite in DMF or benzoquinone in ethanol as oxidizing agents to produce the bis-nitriles **7a-c**. These bis-nitriles were converted to the corresponding diamidines employing the Pinner methodology [15], [16], where the bis-nitriles were stirred in ethanolic HCl(gas) to produce the intermediate imidate ester hydrochlorides that were allowed to react with ethanolic ammonia.

### 2.2 Biology

Aromatic diamidines bind, in part due to their cationic nature, strongly and reversibly with DNA. The mode of the antiprotozoal action of the aromatic diamidines has not been fully determined, but is thought to involve interaction with DNA dependent enzymes or to directly inhibit DNA transcription [17]. The results for assessment of the DNA binding affinity for **4a-d** and **8a-c**, as well as *in vitro* activity against *T. b. r.* STIB900 and *P. f.* K1 and also the *in vivo* activity against *T. b. r.* STIB900 in mice are presented in Table 1. We have previously reported that mesaurment of the increase in melting temperature Δ*Tm* (*Tm* of DNA-ligand complex – *Tm* of free DNA) gives an accurate and fast method for ranking the binding affinities for aromatic diamidines [18]. The Δ*Tm* values for the diamidines/poly (dA-dT) complexes are quiet high ranging from 27 °C to more than 30 °C which demonstrates the strong DNA binding affinity of this class of compounds.

**Table 1:** DNA affinities and antiprotozoal activity for the new diamidines.

	Code	ΔTm (^o^C)^a^	T. b. r. (nM)^b^	P.f. (nM)^c^	Cytotox. (μM)^d^	SI/T. b. r.^e^	SI/P.f^f^	In vivo (cures)^g^
	**4a**	28	15	2.9	16.5	1100	1000	0/4
	**4b**	>30	15	7.3	15.7	1046	2150	0/4
	**4c**	28	4	6.8	17.6	4400	2588	4/4
	**4d**	>30	2	6.8	11.6	5800	1705	0/4
	**8a**	29	102	91.9	41.8	409	454	NA
	**8b**	27	64	NA	15.0	234	NA	NA
	**8c**	>30	25	NA	26.2	1048	NA	NA

a Thermal melting increase of poly(dA-dT)_n_.

b STIB900 was the strain of *T. b. r*. (*Trypanosoma brucei rhodesiense*) used.Values are the average of duplicate determinations.

c IC_50_ values obtained against the chloroquine resistant *P. f.* (*Plasmodium falciparum*) strain. K1. Values are the average of duplicate determinations.

d Cultured L6 rat myoblast cells was used for cytotoxicity assessment.

e Selectivity Index for *T. b. r*. is the ratio: IC_50_ (L6)/IC_50_ (*T b.r*.).

f Selectivity Index for *P. f*. is the ratio: IC_50_ (L6)/IC_50_ (*P.f*.).

g In vivo efficacy determined in *T. b. rhodesiense* (STIB900) infected mice at 4 × 5 mg/kg i.p. dose.

**Fig. 2:**
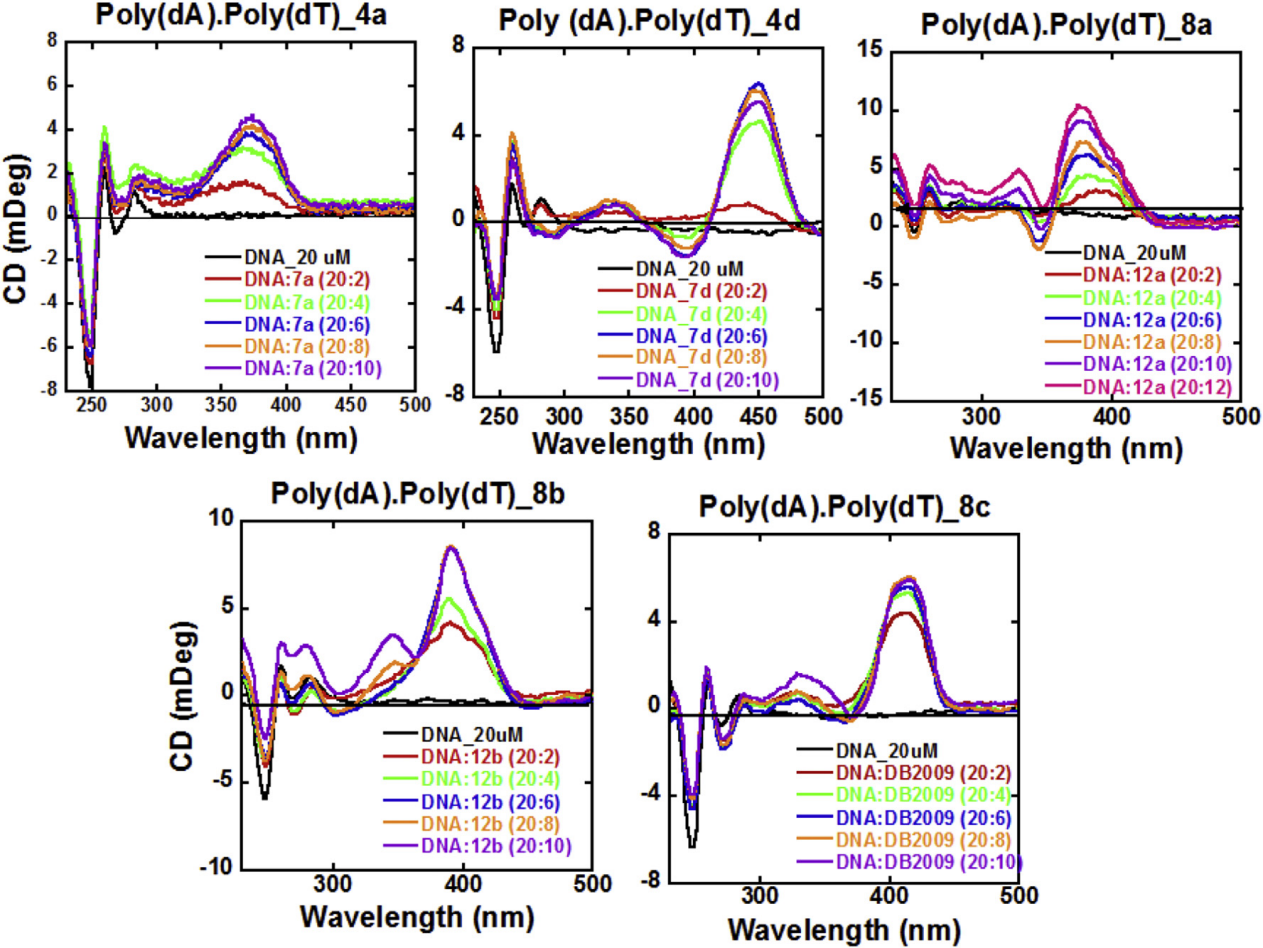
Circular dichroism spectra of the titration of representative compounds, (**4a, 4d, 8a, 8b, 8c**) with a 20 μM Poly(dA).Poly(dT) sequence in the buffer (10 mM MES, 100 mM NaCl, 1 mM EDTA, at pH, 7.4) at 25 °C.

In general, aromatic diamidines are well known to bind in the DNA minor groove at AT base pair sequences [19]. Circular Dichroism (CD) has been found to be a reliable technique for detecting DNA minor groove binding by small molecules. We observed the CD spectra of several of the new diamidines in order to determine if these new bichalcophenes are binding in the DNA minor groove. Minor groove binding compounds show a large positive induced CD signal upon binding to AT DNA sequences and lead to only minor changes in the appearance of the DNA region of the CD spectrum [20]. Fig. 2 shows the CD spectra for the interaction between Poly (dA). Poly (dT) and five of the diamidines namely (**4a, 4d, 8a, 8*b*, 8c**). All five compounds showed very strong induced CD signals which is evidence of a strong interaction with the DNA minor groove, clearly supporting a minor groove binding mode for these diamidines.

The new indole bichalcophenes **4a-d** are highly active against trypanosomes (IC_50_ values from 2 to 15 nM) and are also very active against malaria (IC_50_s from 2.9 to 7.3 nM). These narrow ranges limit SAR evaluations. These indole analogues showed excellent selectivity against both parasites, with selectivity indices from 1046 to 5800 against *T. b. r.* and from 1000 to 2588 against *P. f.* The antitrypanosomal activity of the benzimidazole diamidines **8a-c (**IC_50_ values from 25 to 102 nM) is generally lower than that of the indole analogues **4a-d**. The antimalarial activity of the benzimidazole diamidine **8a** is also only moderate (IC_50_ of 91.9 nM) compared with its indole counterpart **4a** (IC_50_ of 2.9 nM).

The excellent *in vitro* activity and selectivity of the indole bichalcophenes diamidines (**4a-d**) encouraged us to test them *in vivo* in the rigorous *T. b. r*. STIB900 mouse model (Table 1) for the acute hemolymphatic stage of the African trypanosomiasis. The dosage applied was low with a daily intraperitoneal (i.p.) dose of 5 mg/kg compound for four consecutive days.

At this dose pentamidine cures only 1/4 infected mice [21]. The benzimidazole bichalcophenes that were *in vitro* active against *T. b. r*. with IC_50_s > 100 nM have not been tested *in vivo* due to inferior *in vitro* activity and **8b** and **8c** due to termination of the project. Compound **4c** was highly effective *in vivo* with 100% sterile cures in mice infected with *T. b. r*. This excellent activity merits further evaluation of **4c**.

## 3 Conclusion

The newly synthesized dicationic indole- and benzimidazole bichalcophenes exhibited very strong DNA binding as seen from the Δ*Tm* values. Moreover, these diamidines are shown to be DNA minor groove binders as seen from the CD spectra. Both dicationic indole and benzimidazole derivatives showed good antimalarial activity *in vitro*. Both types were also highly active against *T. b. r.* whereas the dicationic indole diamidines were found to be more active than the dicationic benzimidazole derivatives. Specifically, the indole derivative **4c** was highly efficacious *in vivo* curing all *T. b. r.* infected mice at a low dose of 4 × 5 mg/kg i.p. which is more active *in vivo* than the drug pentamidine that has been clinically used to treat African sleeping sickness for more than seven decades.

## 4 Experimental

### 4.1 Biology

#### 4.1.1 Efficacy and cytotoxicity studies

The *in vitro* viability assays were with the *T. b. r*. strain STIB 900 and the chloroquine resistant *P. f*. strain K1. The efficacy studies were with the STIB900 acute mouse model for *T. b. r.* infections [22]. Dosage was 4 × 5 mg/kg i.p. performed as previously described in detail [23]. Cytotoxicity was evaluated in L6 rat myoblast cells as previously reported in detail [24].

#### 4.1.2 Tm measurements

Thermal melting experiments were performed with a Cary 300 spectrophotometer. Cuvettes were mounted in a thermal block where the solution temperatures were mesaured with a thermistor in the reference cuvette. Temperatures were maintained under computer control and increased at 0.5 °C/min. The experiments were performed in 1 cm path length quartz cuvettes in CAC 10 buffer (cacodylic acid 10 mM, EDTA 1 mM, NaCl 100 mM with NaOH added to give pH = 7.0). Poly (dA-dT) DNA concentrations were determined by measuring its absorbance at 260 nm. A ratio of 0.3 mol compound per mole of DNA was used for the complex and DNA alone was used as control [17]. ΔTm values were determined from the peak of first derivative curves (dA/dT).

#### 4.1.3 Circular dichroism (CD)

CD spectra were collected employing a Jasco J-810 spectrometer at 25 °C using different ratios of compound to DNA at 25 °C in MES 10 buffer (10 mM MES, 100 mM NaCl, I mM EDTA).First, a DNA solution in a 1-cm quartz cuvette was scanned over the desired wavelength range. Tested compounds at increasing ratios, were titrated into the same cuvette and the complexes rescanned under the same conditions [25].

### 4.2 Chemistry

All commercial reagents were used without further purification. All melting points were determined on a Mel-Temp 3.0 melting point instrument, and are uncorrected. TLC analysis was carried out on silica gel 60 F254 precoated aluminum sheets using UV light for detection. ^1^H and ^13^C NMR spectra were recorded on a Bruker 400 MHz spectrometer using the indicated solvents. Mass spectra were obtained from the Georgia State University Mass Spectrometry Laboratory, Atlanta, GA. If the compounds are reported as salts contain water and/or solvents, these components were detected in HNMR spectra. Elemental analysis were performed by Atlantic Microlab Inc., Norcross, GA. Compounds **1a-d** [26] and **5a-c** [27] were previously reported.

#### 4.2.1 General procedure for the synthesis of the dinitriles **3a-d**

Tetrakistriphenylphosphine palladium (0.288 g, 0.25 mmol) was added to a stirred solution of the 1-(*tert*-butoxycarbonyl)-2-(trimethylstannyl)-1*H*-indole-6-carbonitrile **2** (2.02 g, 5 mmol) and the bromo derivatives **1a-d** (5 mmol) in air free dioxane (20 mL) under nitrogen. The reaction mixture was stirred at 90–100 °C for 24 h. The reaction mixture was concentrated, the residue was extracted with ethyl acetate (200 mL) containing 5 mL of concentrated ammonia to remove the residual catalyst, dried over sodium sulfate, and evaporated. The residue was chromatographed on silica gel using hexanes/ethyl acetate (85/15, v/v) as solvent.

##### 4.2.1.1 1-(*tert*-Butoxycarbonyl)-2-(5′-cyano-2,2′-bifuran-5-yl)-1*H*-indole-6-carbonitrile (**3a**)

White solid, yield (1.19 g, 60%). mp 221–221.5 °C; ^1^HNMR (CDCl_3_, 400 MHz) δ 8.54(s, 1H), 7.64(d, 1H, *J* = 8.4 Hz), 7.57(dd, 1H, *J* = 1.2 Hz, *J* = 4.0 Hz), 7.52(d, 1H, *J* = 8.4 Hz), 7.30(dd, 1H, *J* = 1.2 Hz, *J* = 4.0 Hz), 7.19(dd, 1H, *J* = 1.2 Hz, *J* = 3.6 Hz), 7.16(dd, 1H, *J* = 1.2 Hz, *J* = 3.6 Hz), 6.78(s, 1H), 1.53(s, 9H); ^13^CNMR (CDCl_3_, 100 MHz) δ 153.2, 149.0, 136.5, 135.5, 135.1, 132.2, 131.8, 129.2, 126.3, 125.2, 125.0, 124.0, 121.4, 120.1, 119.8, 112.1, 111.6, 107.8, 106.3, 85.6, 27.7; ESI-MS: *m*/*z* calculated for C_23_H_17_N_3_O_4_: 399.4, found: 400.3 (M^+^+1); Anal. Calcd. For C_23_H_17_N_3_O_4_: C, 69.17; H, 4.29; N, 10.52. Found: C, 69.02; H, 4.22; N, 10.33.

##### 4.2.1.2 1-(*tert*-Butoxycarbonyl)-2-(5-(5-cyanothiophen-2-yl)furan-2-yl)-1*H*-indole-6-carbonitrile (**3b**)

Yellow solid, yield (1.03 g, 50%). mp 239–240 °C; ^1^HNMR (CDCl_3_, 400 MHz) δ 8.58 (s, 1H), 7.67(d, 1H, *J* = 8.0 Hz), 7.59(d, 1H, *J* = 3.6 Hz), 7.53(dd, 1H, J = 1.2, *J* = 8.0 Hz), 7.28(d, 1H, *J* = 3.6 Hz), 6.91(s, 1H), 6.83(d, 1H, *J* = 3.6 Hz), 6.79(d, 1H, *J* = 3.6 Hz), 1.46(s, 9H); ^13^CNMR (CDCl_3_, 100 MHz) 148.9, 147.9, 146.8, 139.7, 138.2, 136.3, 132.0, 131.7, 126.3, 122.4, 121.7, 120.1, 119.8, 114.1, 113.1, 111.5, 109.6, 108.0, 107.8, 85.4, 27.8; ESI-MS: *m*/*z* calculated for C_23_H_17_N_3_O_3_S: 415.46, found: 416.2 (M^+^+1); Anal. Calcd. For C_23_H_17_N_3_O_3_S: C, 66.49; H, 4.12; N, 10.11. Found: C, 66.55; H, 4.04; N, 9.99.

##### 4.2.1.3 1-(*tert*-Butoxycarbonyl)-2-(5-(5-cyanofuran-2-yl)thiophen-2-yl)-1*H*-indole-6-carbonitrile (**3c**)

Yellow solid, yield (1.01 g, 49%). mp 221–221.5 °C; ^1^HNMR (CDCl_3_, 400 MHz) δ 8.56(s, 1H), 7.65(d, 1H, *J* = 8.0 Hz), 7.53(d, 1H, *J* = 8.0 Hz), 7.41(dd, 1H, J = 0.8, *J* = 3.6 Hz), 7.19(dd, 1H, *J* = 0.8 Hz, *J* = 4.0 Hz), 7.17(dd, 1H, *J* = 0.8 Hz, *J* = 4.0 Hz), 6.81(s, 1H), 6.64(dd, 1H, *J* = 0.8 Hz, *J* = 4.0 Hz), 1.52(s, 9H); ESI-MS: *m*/*z* calculated for C_23_H_17_N_3_O_3_S: 415.46, found: 416.2 (M^+^+1); Anal. Calcd. For C_23_H_17_N_3_O_3_S: C, 66.49; H, 4.12; N, 10.11. Found: C, 66.71; H, 4.27; N, 9.89.

##### 4.2.1.4 1-(*tert*-Butoxycarbonyl)-2-(5′-cyano-2,2′-bithiophen-5-yl)--1*H*-indole-6-carbonitrile (**3d**)

Yellow solid, yield (1.2 g, 56%). mp 253–255 °C; ^1^HNMR (CDCl_3_, 400 MHz) δ 8.55(s, 1H), 7.65(d, 1H, *J* = 8.0 Hz), 7.58(d, 1H, *J* = 4.0 Hz), 7.53(dd, 1H, *J* = 1.2 Hz, *J* = 4.0 Hz), 7.29(d, 1H, *J* = 3.6 Hz), 7.19(d, 1H, *J* = 4.0 Hz), 7.15(d, 1H, *J* = 3.6 Hz), 6.81(s, 1H), 1.53(s, 9H); ESI-MS: *m*/*z* calculated for C_23_H_17_N_3_O_2_S_2_: 431.53, found: 432.2 (M^+^+1); Anal. Calcd. For C_23_H_17_N_3_O_2_S_2_: C, 64.02; H, 3.97; N, 9.74. Found: C, 64.10; H, 3.94; N, 9.68.

#### 4.2.2 General procedure for the synthesis of the diamidine hydrochloride salts **4a-d**

Lithium bistrimethylsilylamide 1M solution in THF (4 mL, 3.98 mmol) was added to a solution of the bis-nitriles **3a-d** (0.66 mmol) in anhydrous THF (5 mL). The reaction mixture was stirred for 2 days at ambient temperature then cooled to zero ^o^C while ethanolic HCl (2 mL) was added. The mixture was allowed to stir for 2 days, anhydrous ether was added and the solid which formed was collected by filtration. The diamidine salt was neutralized with 1*N* sodium hydroxide solution followed by filtration to obtain the free base, that was washed with water and dried. Finally, the free base was converted to the HCl salt through stirring with ethanolic HCl for 5 days at room temperature (to ensure removal of the Boc-group). The ethanolic mixture was diluted with anhydrous ether, and the solid which formed was filtered and dried to give the diamidine salts.

##### 4.2.2.1 2-(5′-Amidino-2,2′-bifuran-5-yl)-1*H*-indole-6-amidine hydrochloride salt (**4a**)

Yellow solid, yield (0.134 g, 61%), mp > 300 °C; ^1^HNMR (DMSO-*d*_6_, 400 MHz) δ 12.75(s, 1H), 9.52(s, 2H), 9.33(s, 2H), 9.16(s, 2H), 9.02(s, 2H), 7.99(d, 1H, *J* = 4.0 Hz), 7.97(s, 1H), 7.77(d, 1H, *J* = 8.4 Hz), 7.48(d, 1H, *J* = 8.4 Hz), 7.36(d, 1H, *J* = 3.6 Hz), 7.32(d, 1H, *J* = 3.6 Hz), 7.17(d, 1H, *J* = 4.0 Hz), 7.05(s, 1H); ^13^CNMR (DMSO-*d*_6_, 100 MHz) δ 166.8, 153.6, 149.6, 149, 148.4, 144.3, 140.5, 136.4, 132.6, 127.3, 121.4, 121.3, 121.0, 119.5, 112.6, 110.7, 109.0, 99.9; ESI-MS: *m*/*z* calculated for C_18_H_15_N_5_O_2_: 333.34, found: 334.2 (amidine base M^+^+1); Anal. Calcd. For C_18_H_15_N_5_O_2_-2.0HCl-1.75H_2_O: C, 49.38; H, 4.71; N, 15.99. Found: C, 49.09; H, 4.92; N, 15.63.

##### 4.2.2.2 2-(5-(5-Amidinothiophen-2-yl)furan-2-yl)-1*H*-indole-6-amidine hydrochloride salt (**4b**)

Yellow solid, yield (0.133 g, 58%), mp > 300 °C; ^1^HNMR (DMSO-*d*_6_, 400 MHz) δ 12.55(s, 1H), 9.44(s, 2H), 9.31(s, 2H), 9.11(s, 2H), 8.96 (s, 2H), 8.14(s, 1H), 7.94(s, 1H), 7.79(d, 1H, *J* = 8.4 Hz), 7.75(d, 1H, *J* = 4.0 Hz), 7.48(d, 1H, *J* = 8.4 Hz), 7.35–7.28(br m, 2H), 6.99(s, 1H); ^13^CNMR (DMSO-*d*_6_, 100 MHz) δ 166.7, 158.8, 148, 147.7, 139.6, 136.4, 135.9, 132.7, 132.6, 127.2, 124.8, 121.4, 121.1, 119.6, 122.5, 112.1, 111.3, 99.8; ESI-MS: *m*/*z* calculated for C_18_H_15_N_5_OS: 349.41, found: 350.2 (amidine base M^+^+1); Anal. Calcd. For C_18_H_15_N_5_OS-2.0HCl-2.3H_2_O-0.1Et_2_O: C, 46.90; H, 4.82; N, 14.86. Found: C, 46.94; H, 4.52; N, 14.47.

##### 4.2.2.3 2-(5-(5-Amidinofuran-2-yl)thiophen-2-yl)-1*H*-indole-6-amidine hydrochloride salt (**4c**)

Yellow solid, yield (0.105 g, 46%), mp > 300 °C; ^1^HNMR (DMSO-*d*_6_, 400 MHz) δ 12.60(s, 1H), 9.45(s, 2H), 9.31(s, 2H), 9.10(s, 2H), 8.98(s, 2H), 7.84–7.91(m, 2H),7.86–7.74(m, 2H), 7.75(d, 1H, *J* = 8.4 Hz), 7.47(d, 1H, *J* = 8.4 Hz), 7.19(d, 1H, *J* = 3.6 Hz), 6.97(s, 1H); ESI-MS: *m*/*z* calculated for C_18_H_15_N_5_OS: 349.41, found: 350.2 (amidine base M^+^+1); Anal. Calcd. For C_18_H_15_N_5_OS-2.0HCl-1.8H_2_O-0.2Et_2_O: C, 48.08; H, 4.83; N, 14.91. Found: C, 47.87; H, 4.50; N, 14.52.

##### 4.2.2.4 2-(5′-Amidino-2,2′-bithiophen-5-yl)-1*H*-indole-6-amidine hydrochloride salt (**4d**)

Yellow solid, yield (0.123 g, 51%), mp > 300 °C; ^1^HNMR (DMSO-*d*_6_, 400 MHz) δ 12.60(s, 1H), 9.44(s, 2H), 9.31(s, 2H), 9.11(s, 2H), 8.96(s, 2H), 8.11(d, 1H, *J* = 4.0 Hz), 7.92(s, 1H), 7.81(d, 1H, *J* = 3.6 Hz), 7.74(d, 1H, *J* = 8.0 Hz), 7.67–7.63(m, 2H), 7.47(d, 1H, *J* = 8.0 Hz), 6.97(s, 1H); ^13^CNMR (DMSO-*d*_6_, 100 MHz) δ 166.8, 158.7, 143.8, 136.6, 136.1, 135.8, 135.0, 134.6, 132.8, 128.3, 127.3, 127.2, 125.7, 121.3, 120.8, 119.6, 112.4, 100.7; ESI-MS: *m*/*z* calculated for C_18_H_15_N_5_S_2_: 365.48, found: 366.2 (amidine base M^+^+1); Anal. Calcd. For C_18_H_15_N_5_S_2_-2.0HCl-1.6H_2_O: C, 46.28; H, 4.35; N, 14.97. Found: C, 46.60; H, 4.18; N, 14.68.

#### 4.2.3 General procedure for the synthesis of the dinitriles **7a-c**

##### 4.2.3.1 First method

1,4-Benzoquinone (1.82 g, 16.9 mmol) was added to a suspension of 3,4-diaminobenzonitrile (1.5 g, 11.25 mmol) and the aldehydes **5a-c** (12.3 mmol) in ethanol (60 mL). The mixture was refluxed under nitrogen for 12 h. The solvent was removed under reduced pressure. Purification was performed using chromatography on silica gel, and hexanes/ethyl acetate (50/50, v/v) as solvent.

##### 4.2.3.2 Second method

Sodium bisulphite (4.3 g, 22.5 mmol) was added to a suspension of 3,4-diaminobenzonitrile (1.5 g, 11.25 mmol) and the aldehydes **5a-c** (12.3 mmol) in dimethylformamide (20 mL). The reaction mixture was refluxed for 8 h under nitrogen. The reaction mixture was poured into water, filtered and the solid was dried. Purification was done using chromatography on silica gel, and hexanes/ethyl acetate (50/50, v/v) as solvent.

##### 4.2.3.3 2-(5′-Cyano-2,2′-bifuran-5-yl)-1*H*-benzimidazole-5(6)-carbonitrile (**7a**)

White solid, yield (1.85 g, 55%). mp 280–282 °C; ^1^HNMR (DMSO-*d*_6_, DMSO-*d*_6_, 400 MHz) δ 13.7(s, 1H), 8.2(s, 1H), 7.82(d, 1H, *J* = 3.6 Hz), 7.72–7.64(m, 2H), 7.49(brs, 1H), 7.32(d, 1H, *J* = 3.6 Hz), 7.19(d, 1H, *J* = 3.6 Hz); ESI-MS: *m*/*z* calculated for C_17_H_8_N_4_O_2_: 300.27, found: 301.1 (M^+^+1); Anal. Calcd. For C_17_H_8_N_4_O_2_: C, 68.00; H, 2.69; N, 18.66. Found: C, 68.02; H, 2.74; N, 18.49.

##### 4.2.3.4 2-(5-(5-Cyanothiophen-2-yl)furan-2-yl)-1*H*-benzimidazole-5(6)-carbonitrile (**7b**)

White solid, yield (2.16 g, 61%). mp 291–293 °C; ^1^HNMR (DMSO-*d*_6_, 400 MHz) δ 13.7(s, 1H), 8.17(brs, 1H), 8.05(d, 1H, *J* = 4 Hz), 7.76 (brs, 1H), 7.74(d, 1H, *J* = 4 Hz), 7.64(d, 1H, *J* = 8 Hz), 7.48(d, 1H, *J* = 3.6 Hz), 7.39(d, 1H, *J* = 3.6 Hz); ^13^CNMR (DMSO-*d*_6_, 100 MHz) δ 149.1, 146.0, 145.9, 145.4, 140.7, 139.1, 139.0, 126.7, 126.0, 125.3, 120.4, 116.1, 115.2, 114.8, 112.1, 107.6, 104.9; ESI-MS: *m*/*z* calculated for C_17_H_8_N_4_OS: 316.33, found: 317.1 (M^+^+1); Anal. Calcd. For C_17_H_8_N_4_OS: C, 64.55; H, 2.55; N, 17.71. Found: C, 64.32; H, 2.57; N, 17.63.

##### 4.2.3.5 2-(5′-Cyano-2,2′-bithiophen-5-yl)-1*H*-benzimidazole-5(6)-carbonitrile (**7c**)

Yellwish white solid, yield (2.57 g, 69%). mp > 300 °C; ^1^HNMR (DMSO-*d*_6_, 400 MHz) δ 13.8(s, 1H), 8.14(brs, 1H), 8 (d, 1H, *J* = 3.6 Hz), 7.93 (brs, 1H), 7.74(brs, 1H), 7.71(d, 1H, *J* = 3.2 Hz), 7.64(d, 1H, *J* = 3.2 Hz), 7.62(s, 1H); ESI-MS: *m*/*z* calculated for C_17_H_8_N_4_S_2_: 332.4, found: 333 (M^+^+1); Anal. Calcd. For C_17_H_8_N_4_S_2_: C, 61.43; H, 2.43; N, 16.86. Found: C, 61.22; H, 2.45; N, 16.85.

#### 4.2.4 General method for the preparation of the diamidine hydrochloride salts **8a-c**

The bis-nitriles **7a-c** (2.1 mmol) were suspended in saturated ethanolic-HCl(gas) and stirred at ambient temperature for 1 week, with complete isolation from air. Anhydrous ether was added and the crystals which formed were filtered, dried under vacuum for 30 min and then suspended in anhydrous ethanol. Ammonia gas was bubbled into the solution for 1 h while keeping the reaction temperature at 0–5 °C and the resulting solution was stirred at room temperature for 4 days. Anhydrous ether was added and the precipitated crystals (HCl salt) were filtered. The final compounds were purified first by neutralization with 1N sodium hydroxide solution followed by filtration of the formed free base which was washed with water and dried. The free base was stirred with ethanolic-HCl(gas) for 2 days, diluted with anhydrous ether, and the solid which formed was filtered and dried to give the diamidine hydrochloride salt.

##### 4.2.4.1 2-(5′-Amidino-2,2′-bifuran-5-yl)-1*H*-benzimidazole-5(6)-amidine hydrochloride salt (**8a**)

Yellow solid, yield (0.45 g, 42%), mp > 300 °C; ^1^HNMR (DMSO-*d*_6_, 400 MHz) δ 9.8(s, 2H), 9.76(s, 2H), 9.74(s, 2H), 9.6(s, 2H), 8.19(d, 1H, *J* = 4 Hz), 8.02(d, 1H, *J* = 4 Hz), 7.84(d, 1H, *J* = 8.4 Hz), 7.76(d, 1H, *J* = 8.4 Hz), 7.71(dd, 1H, *J* = 8 Hz, 4 Hz), 7.32–7.27(m, 1H), 7.21(brs, 1H); ESI-MS: *m*/*z* calculated for C_17_H_14_N_6_O_2_: 334.33, found: 335.2 (amidine base M^+^+1); Anal. Calcd. For C_17_H_14_N_6_O_2_-2.0HCl-2.75H_2_O-0.75Et_2_O: C, 46.88; H, 5.70; N, 16.40. Found: C, 46.59; H, 5.95; N, 16.16.

##### 4.2.4.2 2-(5-(5-Amidinothiophen-2-yl)furan-2-yl)-1*H*-benzimidazole-5(6)-amidine hydrochloride salt (**8b**)

Yellow solid, yield (0.42 g, 39%), mp > 300 °C; ^1^HNMR (DMSO-*d*_6_, 400 MHz) δ 9.5(s, 2H), 9.4(s, 2H), 9.18(s, 2H), 9.08(s, 2H), 8.23–8.15(m, 2H), 7.83–7.81(m, 2H), 7.73(d, 1H, *J* = 8.8 Hz), 7.63(brs, 1H), 7.39(brs, 1H); ESI-MS: *m*/*z* calculated for C_17_H_14_N_6_OS: 350.39, found: 351 (amidine base M^+^+1); Anal. Calcd. For C_17_H_14_N_6_OS-2.0HCl-2.75H_2_O-0.65Et_2_O: C, 45.18; H, 5.41; N, 16.12. Found: C, 45.22; H, 5.70; N, 15.95.

##### 4.2.4.3 2-(5′-Amidino-2,2′-bithiophen-5-yl)-1*H*-benzimidazole-5(6)-amidine hydrochloride salt (**8c**)

Yellow solid, yield (0.37 g, 37%), mp > 300 °C; ^1^HNMR (DMSO-*d*_6_, 400 MHz) δ 9.49(s, 2H), 9.39(s, 2H), 9.27(s, 2H), 9.18(s, 2H), 8.15–8.13(m, 3H), 7.79–7.70(m, 4H); ESI-MS: *m*/*z* calculated for C_17_H_14_N_6_S_2_: 366.46, found: 367 (amidine base M^+^+1); Anal. Calcd. For C_17_H_14_N_6_S_2_-3.0HCl-0.5H_2_O: C, 42.11; H, 3.74; N, 17.33. Found: C, 41.98; H, 4.02; N, 17.03.
