# Supplementary figures and images for: Indole and Benzimidazole Bichalcophenes: Synthesis, DNA Binding and Antiparasitic Activity
Source: Eur J Med Chem. 2018 Jan 1;143:1590–6. doi: 10.1016/j.ejmech.2017.10.056 (PMC5744864; doi:10.1016/j.ejmech.2017.10.056)

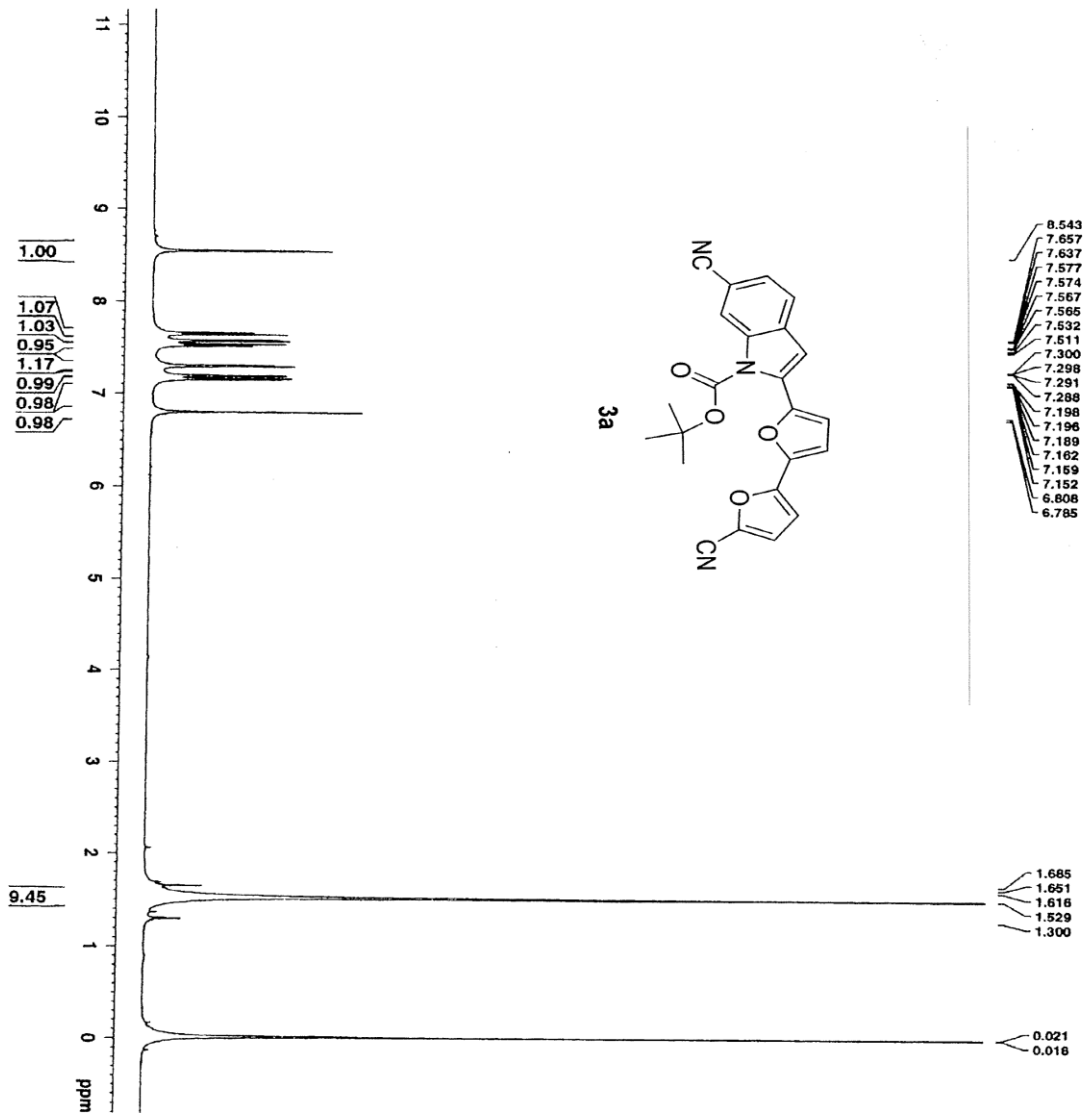

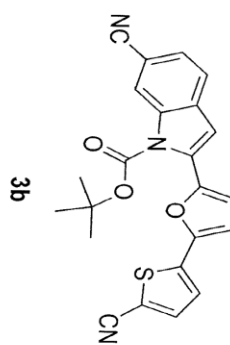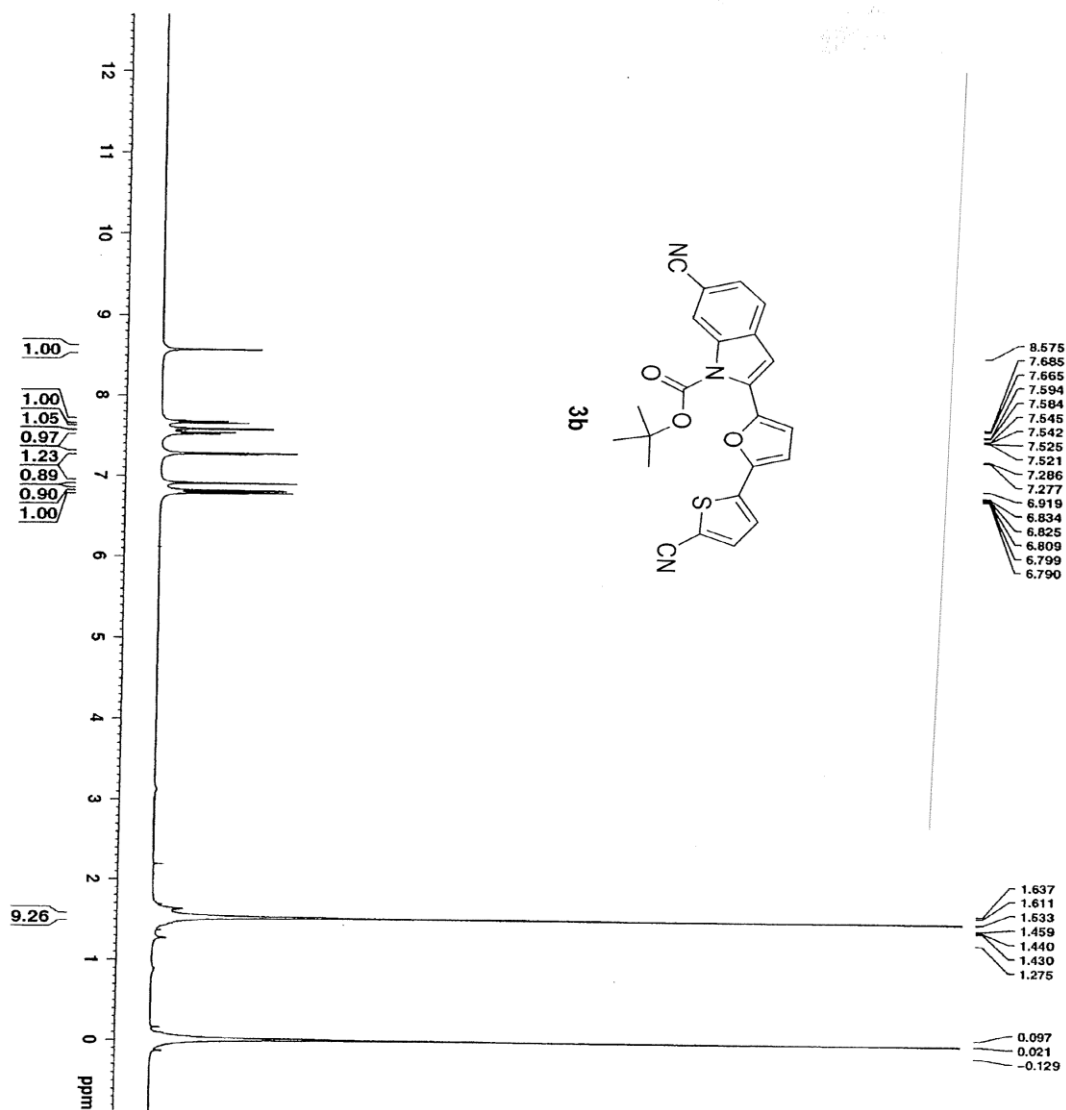

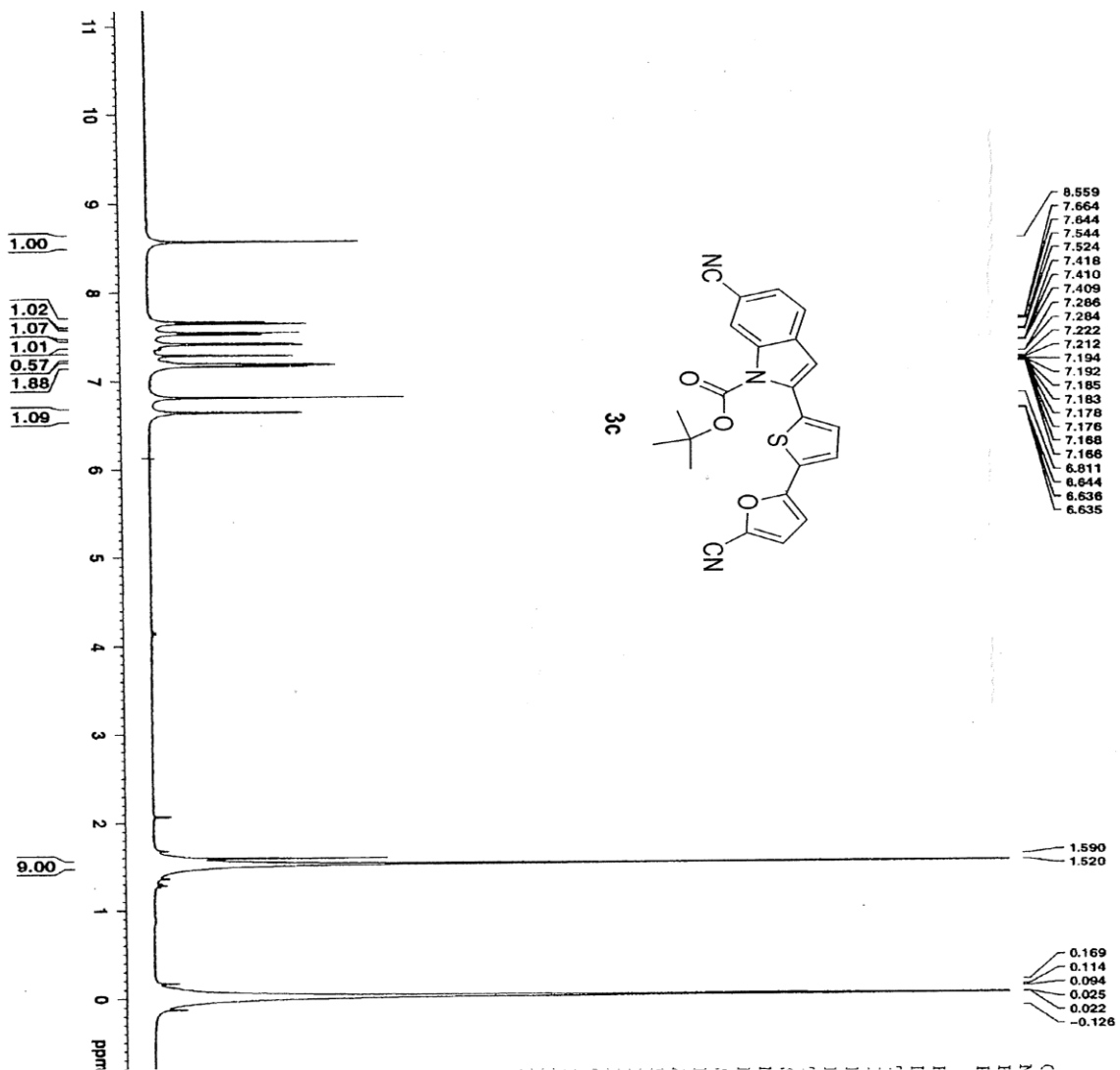

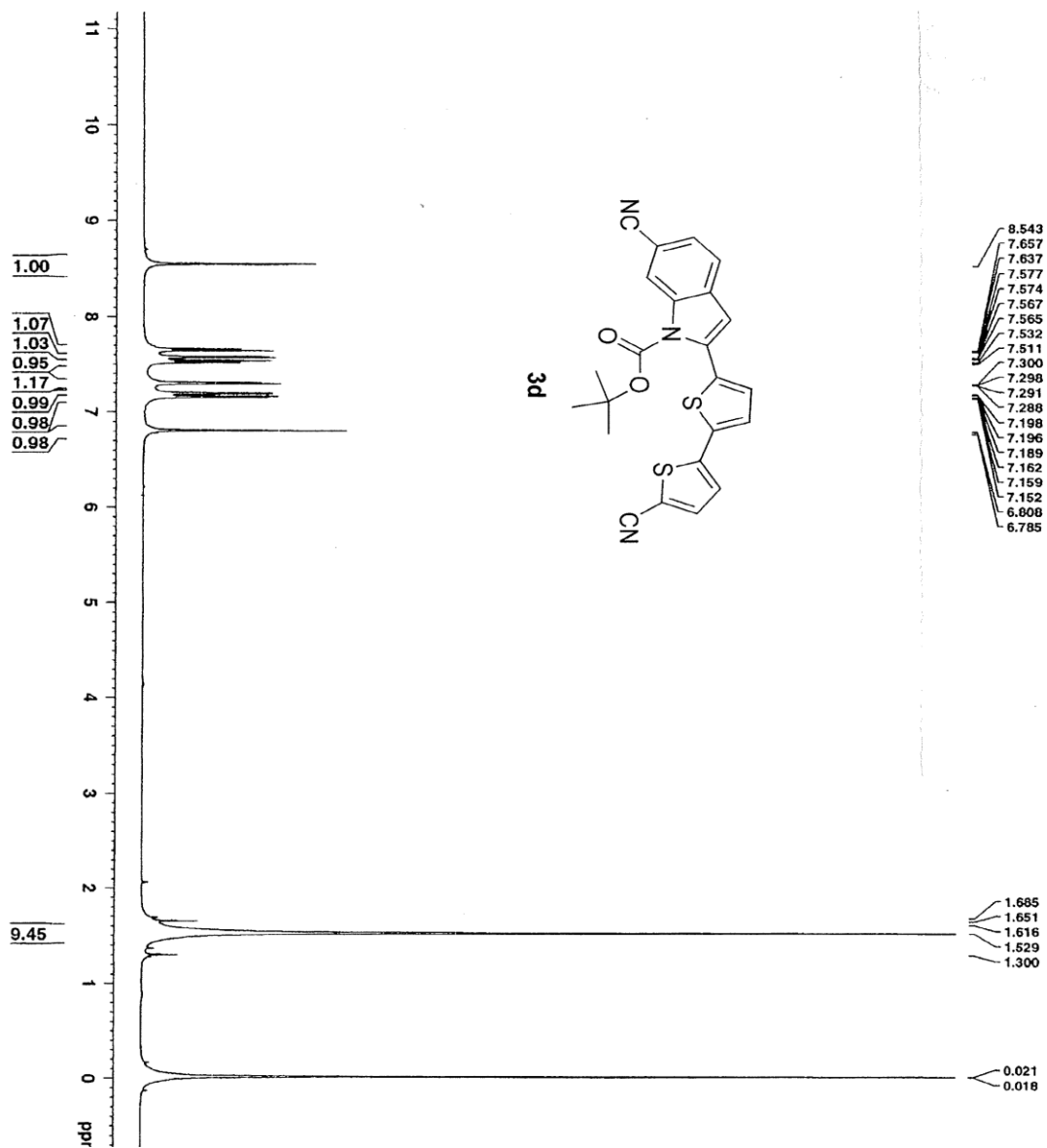

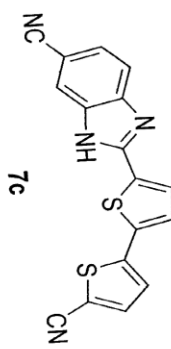

7.641  
7.633

3.381

2.511

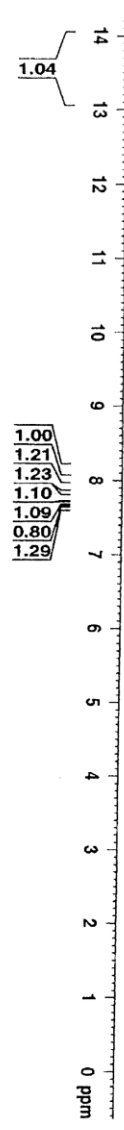

9.521  
9.337  
9.163  
9.022  
7.995  
7.986  
7.970  
7.788  
7.767  
7.490  
7.469  
7.359  
7.350  
7.319  
7.310  
7.178  
7.168  
7.050

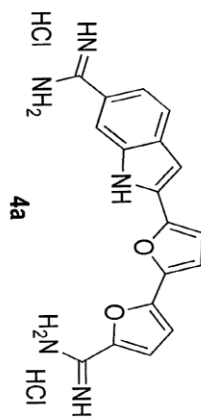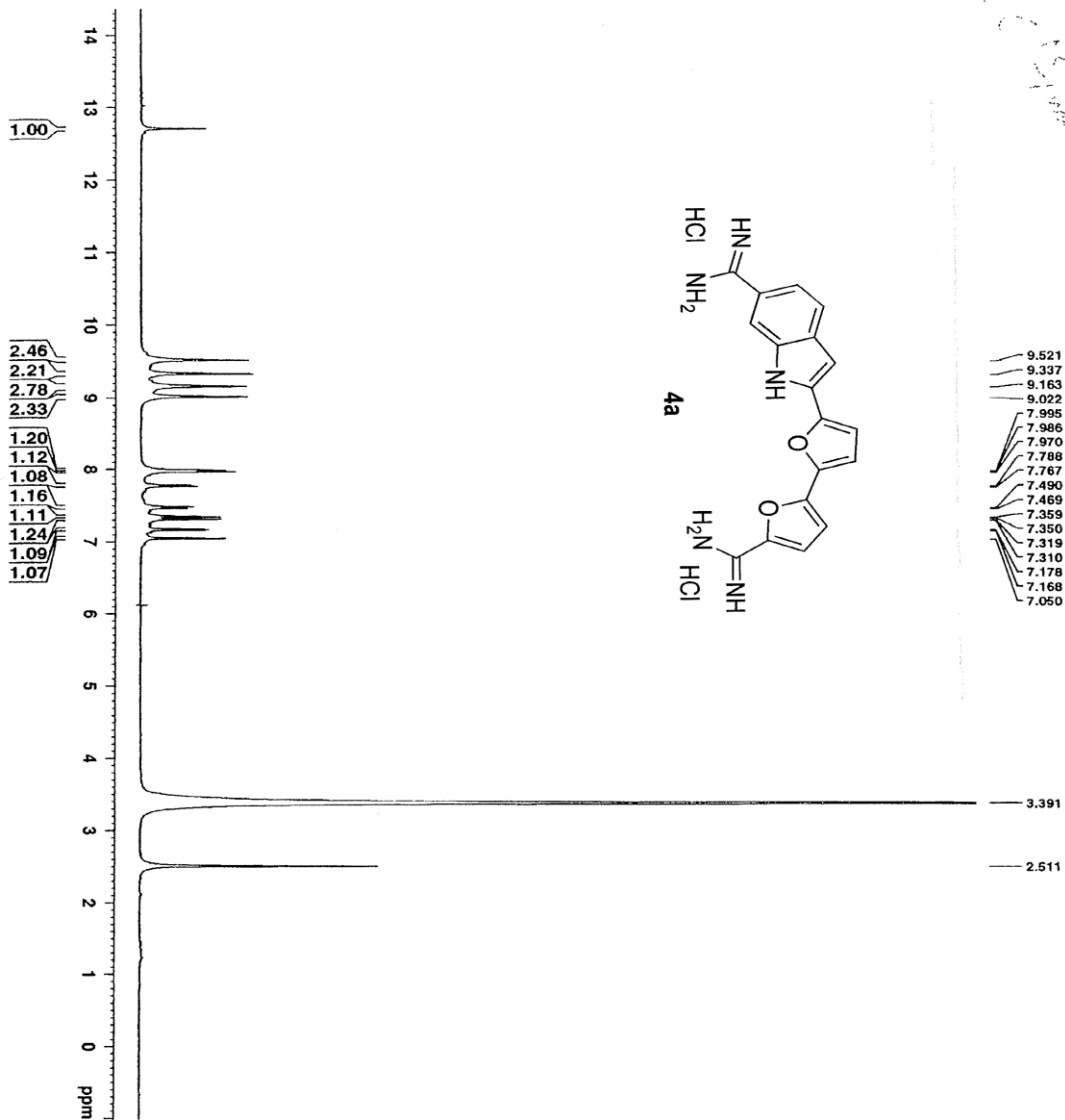

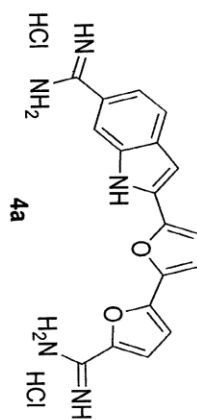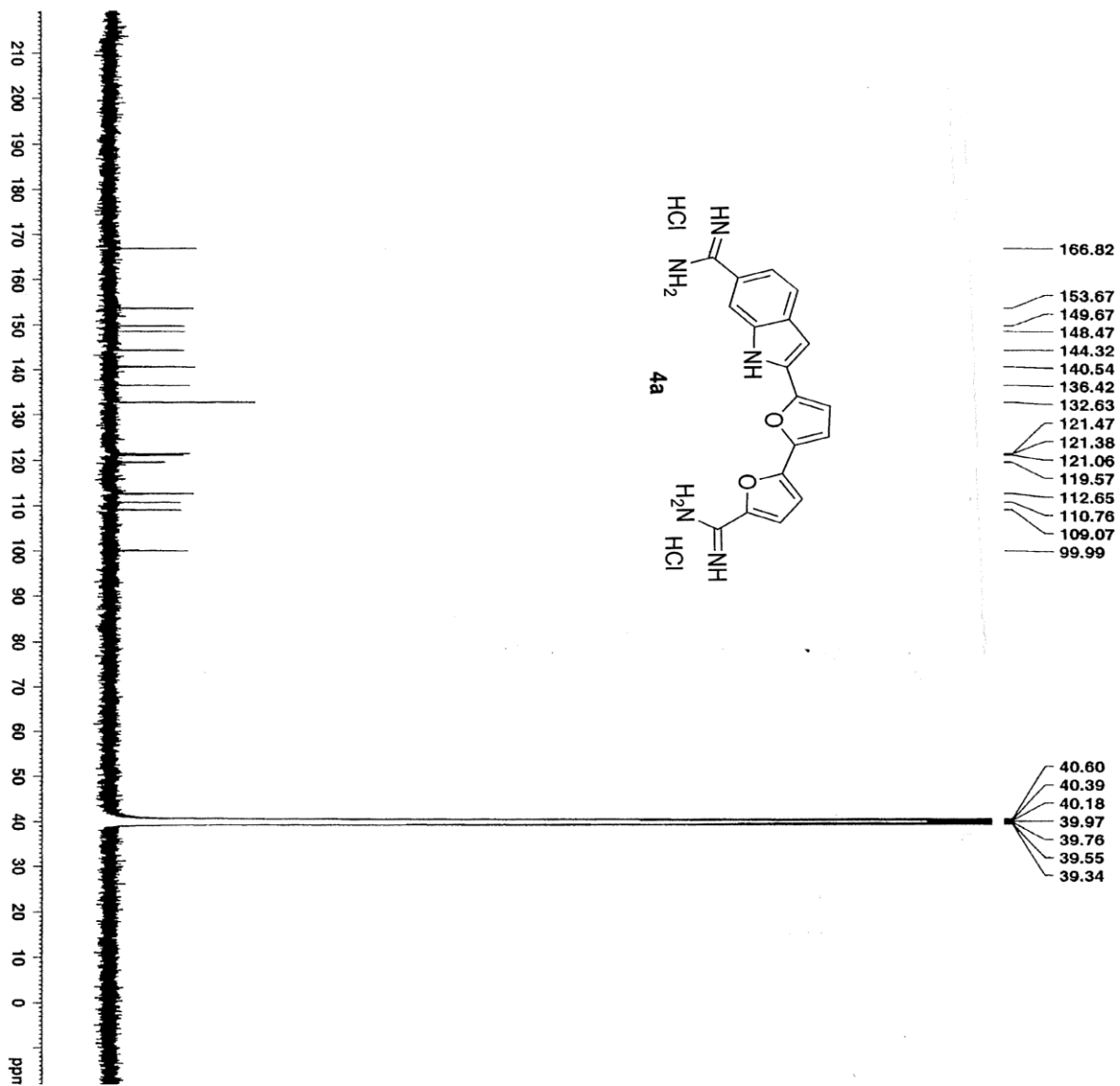

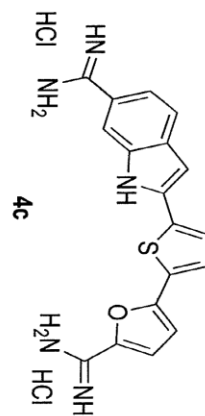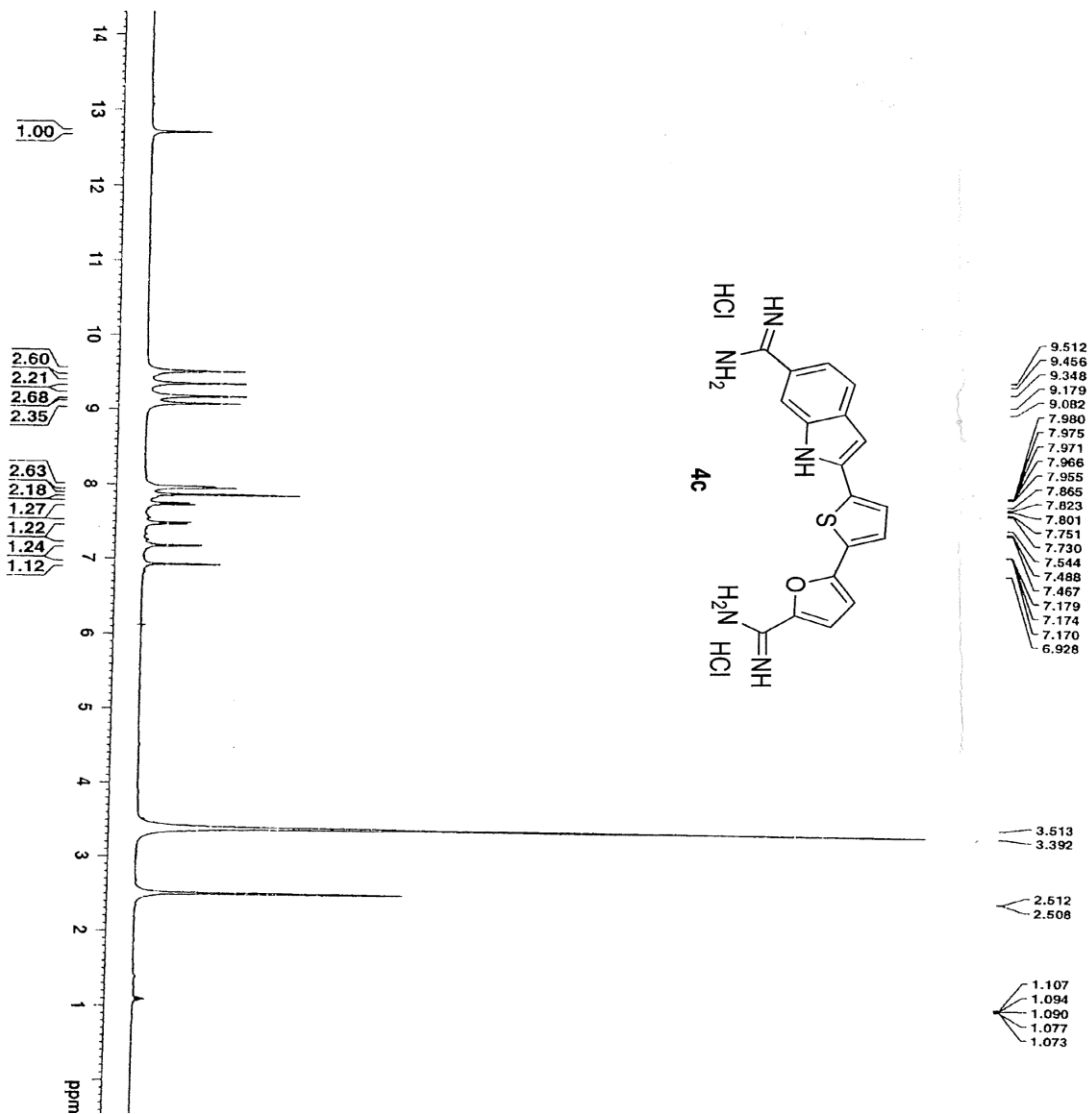

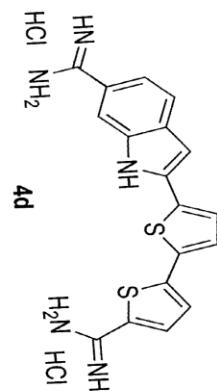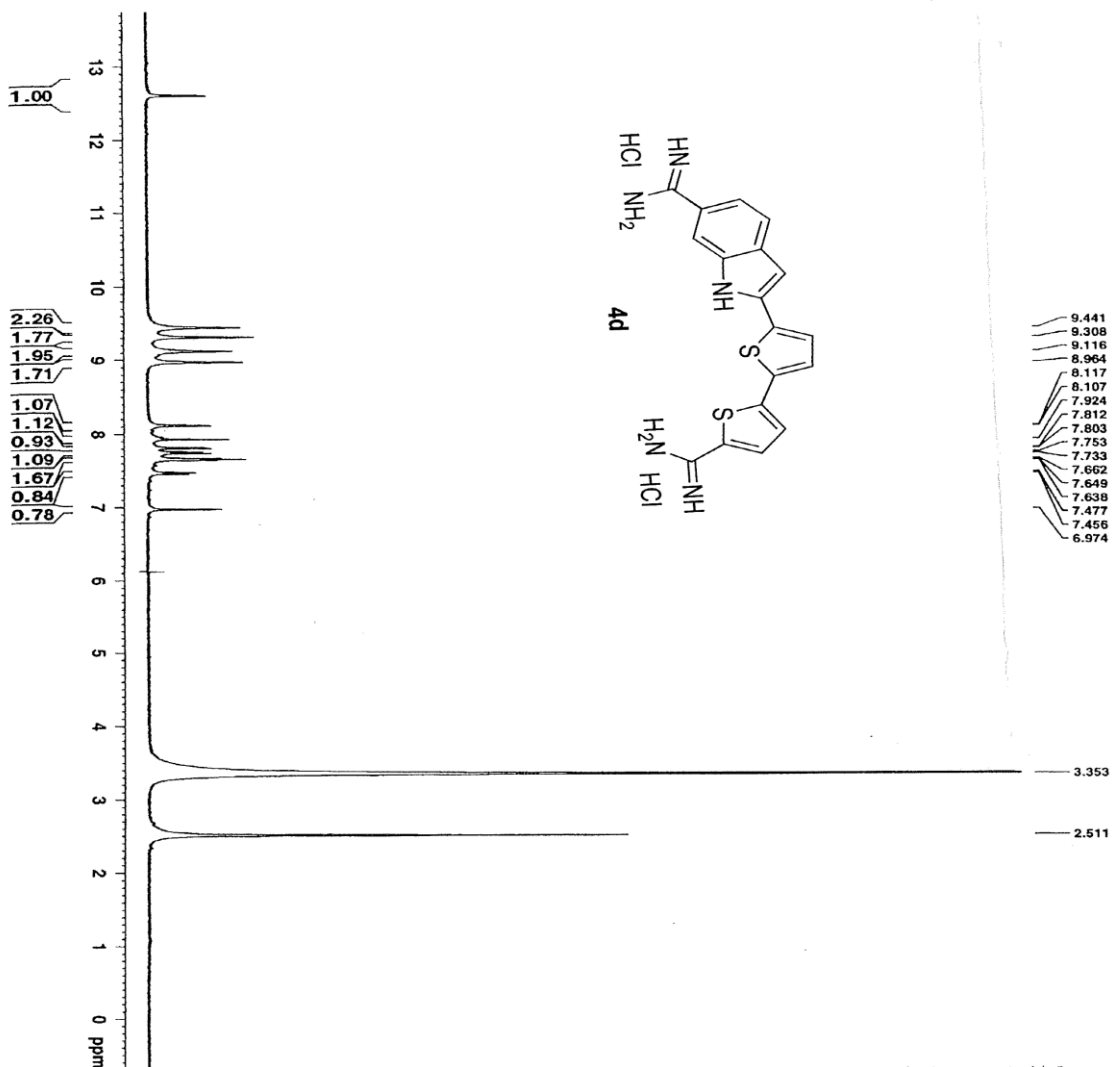

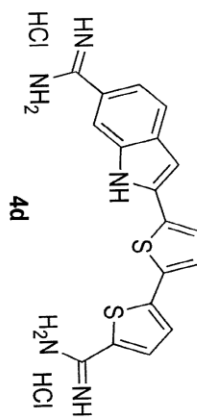

4d

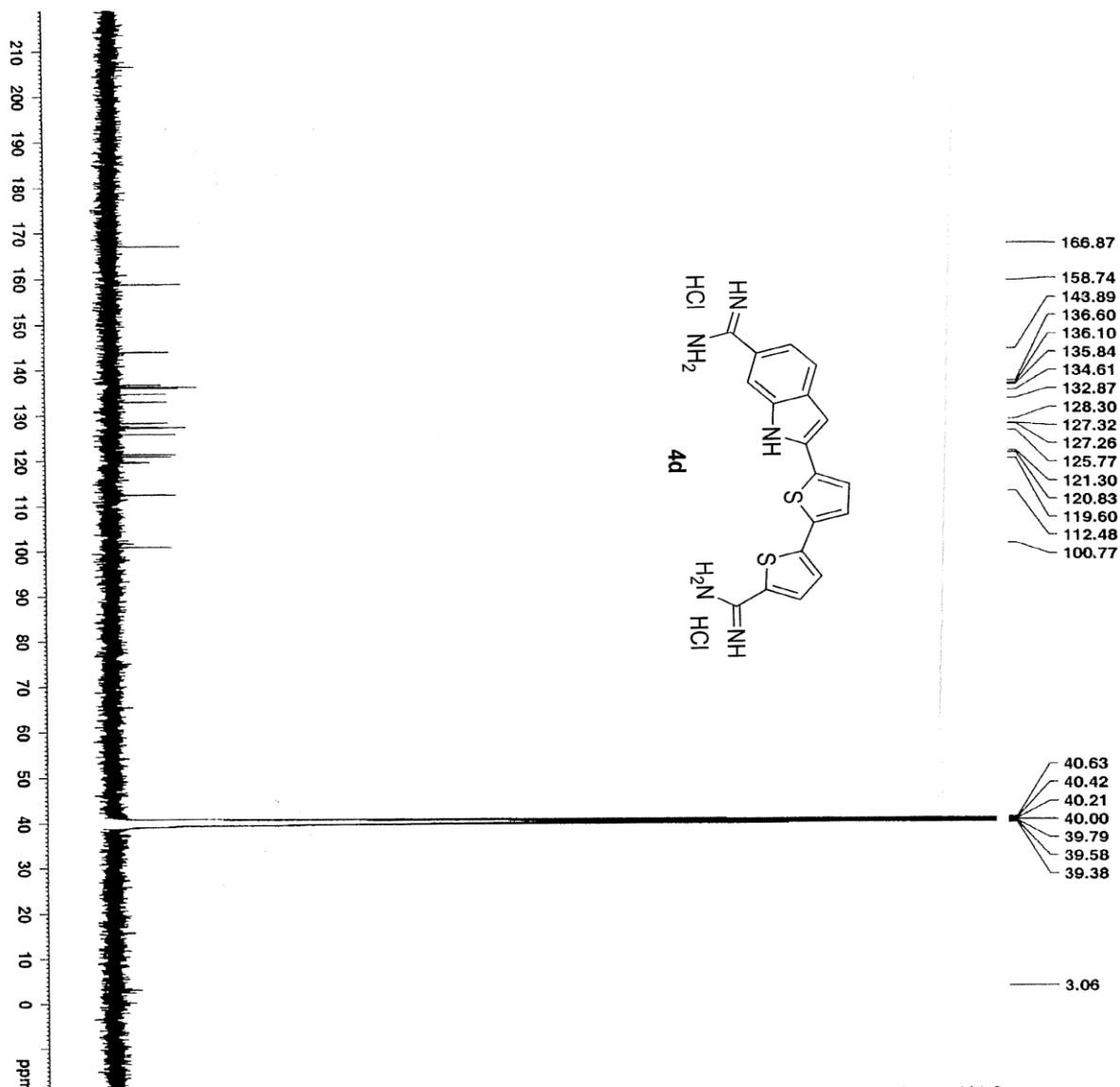

✓

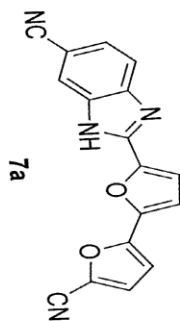

8.209  
7.824  
7.614  
7.773  
7.727  
7.647  
7.491  
7.328  
7.319  
7.198  
7.189

3.341  
2.512

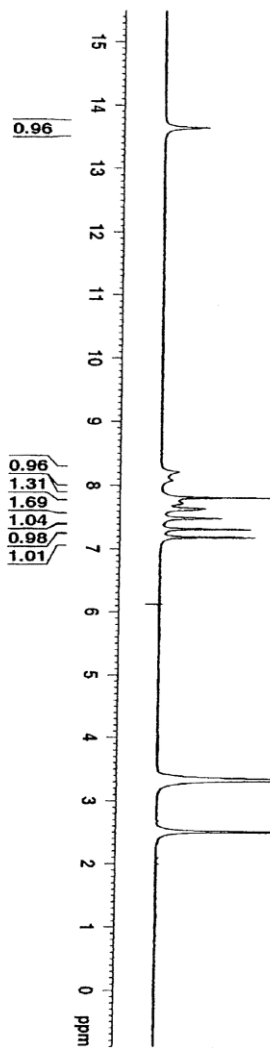

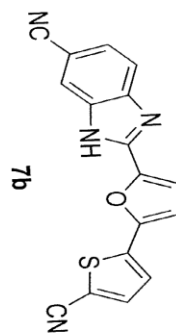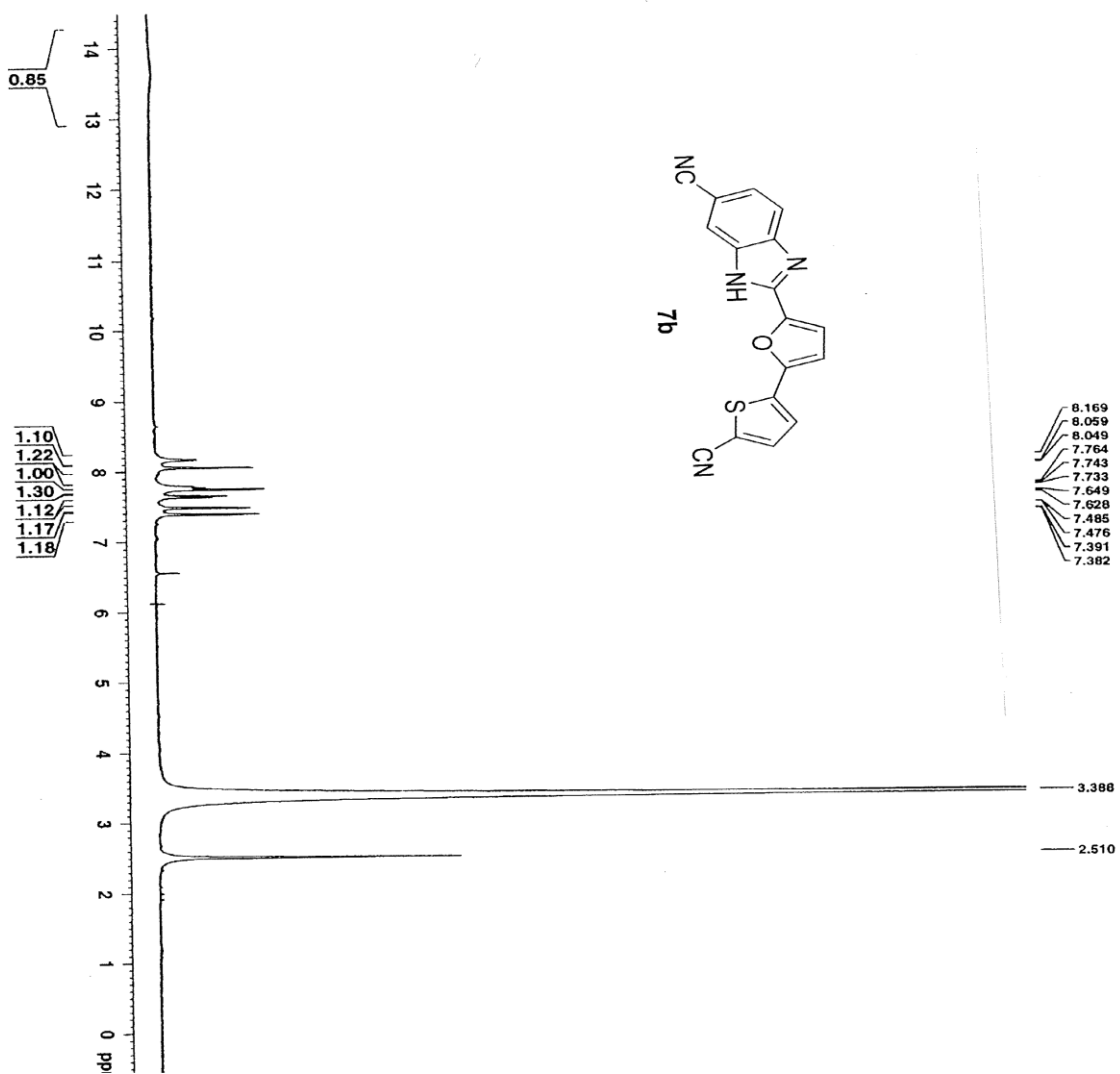

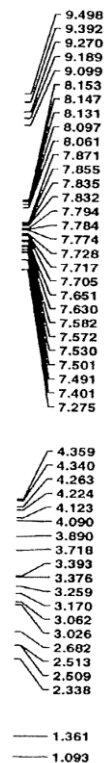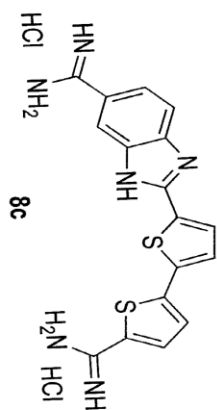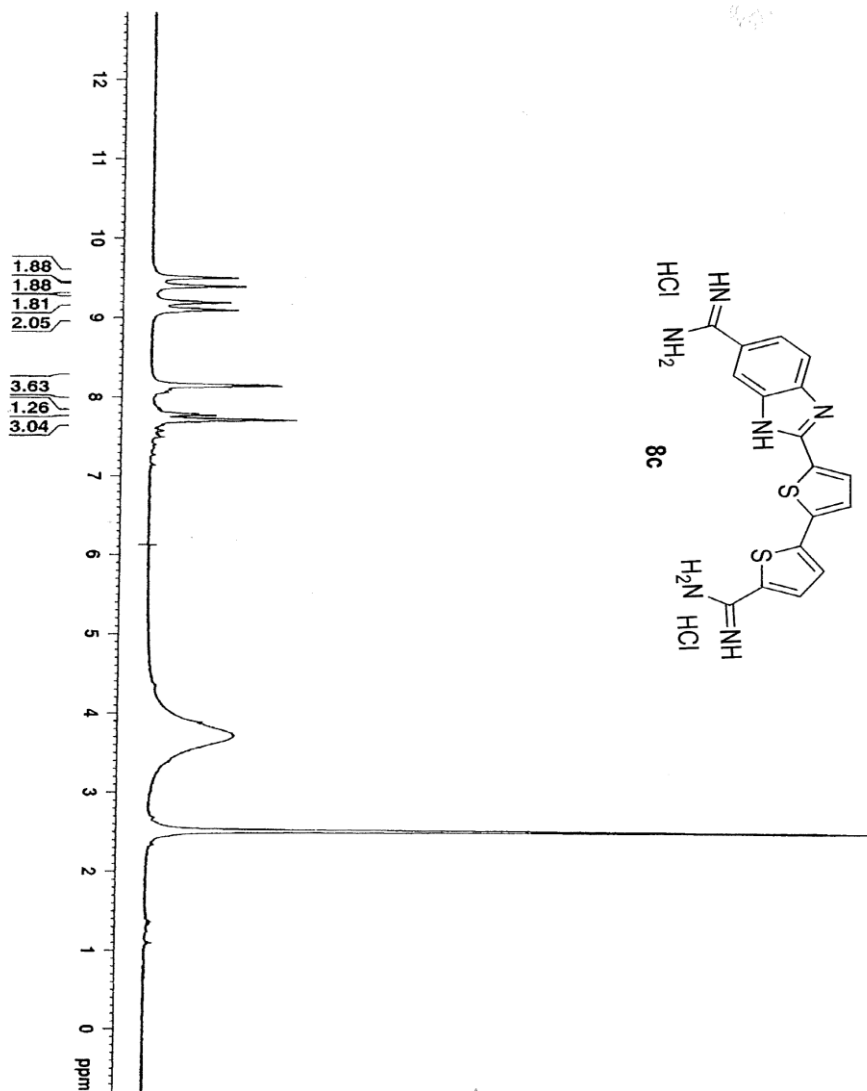

Supplement: Supplementary data [file mmc1.pdf]
